# Evaluating the impact of a community-based prostate cancer screening program

**DOI:** 10.1186/s13690-025-01779-x

**Published:** 2025-12-24

**Authors:** Amy E. Leader, Kristine Pham, Nicola Cianci, Hannah Knapp, Fatuma Doka, Suzanne Barron, Oleksandr Kolesnikov, Jordan DeHoratius, Katie Bradley, Le’Ann Robles, Robert Giamboy, Olivia Dahlgren, Leonard G. Gomella

**Affiliations:** 1 Department of Medical Oncology, Sidney Kimmel Medical College, Thomas Jefferson University, Philadelphia, PA USA; 2 Sidney Kimmel Comprehensive Cancer Center, Thomas Jefferson University, Philadelphia, PA USA; 3 Carleton College, Northfield, MN USA; 4 Department of Urology, Sidney Kimmel Medical College, Thomas Jefferson University, Philadelphia, PA USA

**Keywords:** Prostate cancer, Cancer screening, Community engagement, Cancer disparities

## Abstract

**Background:**

One out of eight men in the U.S. will be diagnosed with prostate cancer during his lifetime. Black men are more likely to be diagnosed with and die from prostate cancer than other males, contributing to significant prostate cancer disparities. Early detection, often by routine screening, allows for more options for treatment and an increased chance of survival. Barriers to screening contribute to prostate cancer disparities, particularly among those who lack regular access to health care. Community based screening programs may overcome barriers to prostate cancer screening.

**Methods:**

An urban, NCI-designated cancer center collaborated with a national prostate cancer advocacy organization to provide free screening to males in our catchment area at local community organizations. Males who were eligible for prostate screening completed a survey about their personal and family history of prostate cancer. A mobile phlebotomist conducted the screenings, consisting of prostate specific antigen (PSA) tests, onsite at the partner agency. Males received a letter with their PSA test results; males with an abnormal result (> 2.5 ng/mL) also received a phone call. Survey data was analyzed with descriptive statistics and bivariate analyses to assess relationships with PSA test results.

**Results:**

Two hundred and eighty-nine (*n*=289) males were screened at a community screening event. The mean age of males screened was 57.3 (SD=11.2) and the majority identified as Black (*N*=253, 84.9%). The mean PSA test value was 1.82 (SD= 2.70); 18% of males had an abnormal PSA test result. Black males had the highest PSA values of among all men who were screened.

**Discussion:**

Disparities in prostate cancer, particularly among Black males, warrant interventions like community-based prostate screening events that reduce barriers of cost and access, bringing screening to those who would most benefit.

**Table:** 

Text box 1. Contributions to the literature
• Longstanding racial and ethnic disparities in prostate cancer incidence and mortality necessitate novel initiatives, such as community-based screening, to overcome barriers to accessing the health care system.
• This manuscript describes the process of establishing a community-based prostate cancer screening program, which could be adapted or replicated by other agencies or organizations.
• The results of this screening initiative, that Black males had higher PSA values than other males who were screened, adds to the literature that Black males are at increased risk for prostate cancer.

## Background

### Prostate cancer inequities and risk factors

Prostate cancer is the second most common cancer in men, following skin cancer, and the second leading cause of cancer death in males, following lung cancer [1]. Certain populations are at increased risk of prostate cancer incidence and mortality, driven by a combination of genetic, biological, behavioral, environmental, and social factors [2]. Some risk factors are modifiable, such as smoking and chemical exposure; others, such as one’s age, genetic profile, and race/ethnicity, are not. Differences among populations in awareness, screening practices, treatment options, and adherence contribute to prostate cancer disparities [3]. In the United States, Black males face a mortality rate from prostate cancer that is almost twice as high as white males [4].

### Screening guidelines and rates of screening

For males aged 55 to 69 years, the United States Preventive Services Task Force recommends that men discuss the potential benefits and harms of prostate cancer screening with their clinician, to make an informed decision about screening [5]. Other organizations recommend that males at increased risk, such as those who are Black or have a family history of prostate cancer, start screening earlier at age 40 or 45 [6–8]. Recent national estimates suggest that approximately 37% of age-eligible individuals have had a prostate specific antigen (PSA) test, the primary form of screening, in the last year; only 33% of Black males and 29% of Hispanic males were screened, compared to 41% of White males [9]. The disparity in screening rates can be attributed to a multitude of factors, among which include mistrust of the medical system, lack of awareness or confusion about the importance of screening, and lack of provider recommendation for PSA screening [10]. Access to a primary care provider has also been shown to predict routine screening, demonstrating the disparity among those without regular access to care or uninsured individuals [11].

In recent years there has been uncertainty about the effectiveness and risk associated with PSA screening, consequentially lowering the rates of prostate cancer screening and increasing confusion surrounding the PSA test [12, 13]. Although there are risks associated with PSA screening such as an unnecessary biopsy or undue mental distress due to a baseline elevated PSA, data suggest that routine screening decreases mortality rates and should be encouraged for those considered at high risk, (i.e. Black men) or those with a family history of prostate cancer [14]. Recent statistical modeling showed that prostate cancer screening contributed to 56% of averted prostate cancer deaths [15].

### Community based prostate cancer screening programs

Community-based screening programs are an effective way to bring cancer screening to communities that lack accessible health care [16]. Evidence suggests that community screening programs should employ multicomponent intervention strategies such as convenient and accessible screening locations that overcome transportation barriers, culturally tailored messages with trusted community leaders, and adequate follow-up, navigation, and connection to care. Partnerships with local organizations leverage community assets, promote trustworthiness, and extend the reach of the health system [17, 18]. Community based cancer screening programs have shown to be effective in breast, cervical, colorectal and lung cancer screening; [19] fewer community-based programs for prostate cancer screening have been implemented and evaluated in the U.S [20].

### The local context for community based prostate cancer screening

The Sidney Kimmel Comprehensive Cancer Center (SKCCC) at Jefferson is an NCI-designated cancer center located in the Greater Philadelphia region. The cancer center serves a 7-county catchment area consisting of Philadelphia County and the 6 surrounding counties in both Pennsylvania and New Jersey. This is a diverse, urban region of the country where more than 40% of the 6 million residents are of a racial or ethnic minority background, with 19% identifying as Black/African American, 11% identifying as Hispanic or Latino, and 6% identifying as Asian [21]. According to the Centers for Disease Control and Prevention, the incidence and mortality rates for prostate cancer are higher for the catchment area than the U.S. (incidence: 130 cases per 100,000 males in the catchment area vs. 116 cases per 100,00 males nationally; mortality: 19.4 cases per 100,000 males in the catchment area vs. 18.9 cases per 100,000 males) [22].

The SKCC Office of Community Outreach and Engagement (COE) is home to its Mobile Cancer Screening Program (MCSP) that aims to provide equitable, community-based cancer screening and follow-up care to the residents within the SKCCC catchment area. The SKCCC MCSP consists of full-time staff dedicated to community connections, patient registration, and nurse navigation. Since November 2022, the SKCC MCSP has partnered with the Department of Urology at Jefferson and the Prostate Conditions Education Council (PCEC) [23] to provide free prostate cancer screening to males living in the Greater Philadelphia region. Here, we describe the development of the program and the role of community partnerships, as well as an evaluation of 289 males who were screened through the program. This community-based screening protocol was approved by the Institutional Review Board at Thomas Jefferson University.

## Methods

### Community partnerships

Each community-based prostate cancer screening event is held in collaboration with a community partner. The SKCCC Office of COE has an extensive network of partnerships with local community organizations spanning the catchment area, such as places of worship, social service agencies, employers, community centers, local departments of health, and more. Typically, a community partner reaches out to the SKCC MCSP to express their interest in hosting a prostate cancer screening event for the males of their community. The partnership with a community organization ensures that screenings are received in a manner that is trustworthy and credible; it also ensures that there is a physical location for the screening event in a neighborhood-based setting. An initial conversation between the SKCC MCSP and the community partner ensures that the screening event will be culturally tailored to the community, whether by providing on-site interpreter services for languages other than English or by understanding processes or customs that are unique to certain communities.

### Screening event logistics

In partnership with PCEC, screening is offered as part of a research protocol, which allows the SKCC MCSP to ask each male to complete a survey prior to the PSA test. The survey was created by PCEC and is comprised of 10 questions related to one’s personal and family history of prostate cancer, as well as demographic characteristics. After a member of the SKCCC MCSP obtains verbal informed consent, the male completes the survey. The survey is available in English, Spanish, and Chinese. Upon completion of the survey, a member of the urology team initiates a discussion with the patient about their decision to undergo PSA testing. This discussion ensures that each patient understands the risks and benefits of screening. Males who elect to participate in PSA screening have their blood drawn by a certified phlebotomist who is part of the SKCCC MCSP. Blood serum is separated by centrifugation and samples are sent to PCEC to be analyzed. Roughly two weeks after the event, PCEC returns the PSA results via secure email to the SKCCC MCSP, where a Jefferson urologist and the SKCCC MCSP’s oncology nurse navigator review the results and confirm abnormal screening results. For community-based screenings, the urologist determined that a conservative value of 2.5 ng/ml is considered an abnormal PSA value. Every patient receives a letter in the mail from the SKCCC MCSP with their results, information about the importance of routine screening, and how to schedule additional medical care at our health system. Males with abnormal results also receive a telephone call from a urology team member to schedule an appointment if they wish to return to our health care system for follow-up care.

### Data management and analysis

Participant survey data and PSA test results are entered by the SKCCC MCSP into a REDCap database, which also serves as the main patient record database for the screenings. This study report results from screening events from April through June 2024. Only patients with complete data were included in analyses.

Four new variables were created by recoding the primary data. We created a measure called “Personal Chronic Disease Burden by assigning a value of “1” to each of the chronic diseases and other health issues indicated by a participant [high cholesterol, high blood pressure, prostate infection, enlarged prostate, prostate surgery, erectile dysfunction or sexual problems, heart attack or stroke, heart disease, type I diabetes, type II diabetes, depression, and prostate cancer] which were summed to create an additive measure of chronic disease burden. We created a measure called “Family History of PSA” by calculating the total number of males in the participant’s family who had been diagnosed with prostate cancer. We created a measure called “Family Genetic Risk History” by calculating the total number of family members with inherited genetic risk factors such as breast cancer, ovarian cancer, and Lynch syndrome. Lastly, we created a variable called “Family Chronic Disease Burden” by adding the total number of family members with a chronic disease from the list above.

Data was exported from REDCap and cleaned and analyzed using IBM SPSS software version 20.0.2.0. Mean scores and frequencies were used to describe the population. One-way ANOVAs were used to understand differences in PSA values by demographic, behavioral, and family history characteristics; in these cases, *p* < 0.05 denoted statistical significance.

## Results

During the period of April through June 2024, we held eight community screening events. Two events were with churches, two events were with fire departments, two events were with community centers, one was at a men’s health festival, and one was at an employer. The events screened various numbers of males, with the lowest being one male screened at a community event to 180 males screened at a church hosting a men’s health event. All events were within our cancer center’s catchment area, considered to be the seven counties comprising the Greater Philadelphia region.

Two hundred and eighty-nine (*n* = 289) males were screened at a community screening event. The mean age of males screened was 57.3 (SD = 11.4) and the majority identified as Black (*N* = 253, 84.9%). The mean PSA test value was 1.82 (SD = 2.70); 18.2% of males had a PSA test result greater than 2.5 ng/mL. Only 46% males (*N* = 138, 46.3%) had a PSA test in the past three years; the remainder either never had a PSA test (*N* = 122, 40.9%) or reported that their last PSA test was more than three years ago (*N* = 38, 12.8%). Sixty-seven males (22.6%) were current or previous smokers and 51 (17.1%) had been exposed to cancerous chemicals in their occupation. More than two-thirds of the males who were screened (*N* = 192, 64.4%). had one or more chronic diseases. Roughly one-quarter of males (*n* = 78, 26.2%) males had one or more family members with a history of prostate cancer and 63 males (21.1%) had one or more family members with a cancer that has a known genetic link to prostate cancer. Further details can be found in Table 1.

**Table 1:** Description of participant characteristics

Participant Characteristics	*N* = 298	%
Gender, Male	298 (100%)	100
Age, Years, Mean (SD)	57.3 (11.2)	
Race/Ethnicity		
Black	253	84.9
Hispanic	18	6.0
White	10	3.4
Other/Missing	17	5.7
Diet, fat intake		
Low	60	20.1
Medium	193	64.8
High	30	10.1
Missing	15	5.0
Smoking status, number of years since quitting		
<10 years	10	3.4
11–19 years	8	2.7
>20 years	22	7.4
Never smoker	202	67.8
Current smoker	27	9.1
Missing	29	9.7
Occupational cancer chemical exposure		
Not exposed	216	72.5
Exposed	51	17.1
Missing	31	10.4
Personal chronic disease burden, number of conditions		
0	106	35.6
1–2	144	48.3
3	48	16.1
Personal history of prostate cancer		
Yes	8	2.7
No	290	97.3
PSA in last 3 years, number of PSA tests		
0	122	40.9
1–3	138	46.3
4–6	7	2.3
7+	5	1.7
Missing	26	8.7
Family history of PCa, number of relatives w/prostate cancer		
0	220	73.8
1	64	21.5
2+	14	4.7
Family history of genetic risk, number of relatives		
0	235	78.9
1	53	17.8
2+	10	3.4
Family chronic disease burden, number of relatives		
0	96	32.2
1–2	124	41.6
3+	78	26.2

Differences among PSA values were observed among populations. Black men had the highest mean PSA level among all males, although the difference was not statistically significant (Black males, *M* = 1.85; White males, *M* = 1.48; Hispanic males, *M* = 0.80; *p* = 0.42). The family history of prostate cancer was significantly associated with PSA level, such that males with no family history of prostate cancer (*M* = 1.78) and males with one family member with a history of prostate cancer (*M* = 1.54) had a significantly lower average PSA level than those with 2 or more family members with a history of prostate cancer (*M* = 3.84) (*p* = 0.027). There were no significant differences among males by personal smoking history, family history of PCA, or chemical exposure. Full results can be viewed in Table 2.

**Table 2:** Mean PSA test results by patient characteristics

	*N*	Mean PSA Level	Sum of Squares, DF		F	*p*
			Between Groups	Within Groups		
Full Sample	298	1.82				
Race/Ethnicity			3; 19.62	271; 1876.18	0.94	0.42
Black	246	1.85				
Hispanic	18	0.80				
White	9	1.48				
Other	2	1.59				
PSA in the last 3 years, # tests			3; 19.43	260; 1861.65	0.91	0.44
0	121	1.62				
1–3	133	1.82				
4–6	6	3.40				
7+	4	2.08				
Family history of PCa, # of relatives			2; 54.09	287; 2051.72	3.78	0.024*
0	215	1.78				
1	63	1.50				
2+	12	3.84				
Family history of PCa genetic risk, # of relatives			2; 11.75	287; 2094.06	0.81	0.45
0	229	1.71				
1	52	2.24				
2+	10	1.96				
Smoking status			4; 31.74	256; 1815.53	1.12	0.35
Current	26	2.19				
Former, quit within 10 years	8	0.93				
Former, quit within 11–19 years	8	1.46				
Former, quit more than 20 years ago	20	2.75				
Never	199	1.68				
Diet, fat intake			2; 9.49	272; 1888.09	0.68	0.51
Low	60	1.65				
Medium	186	1.71				
High	29	2.29				
Chemical Exposure			1; 2.80	257; 1824.87	0.39	0.53
Not exposed	210	1.72				
Exposed	49	1.97				

*Indicates significance (*p* < 0.05)

## Discussion

Community based screening programs are valuable in reducing barriers and increasing access to cancer screenings. While recognized as an evidence-based approach to cancer screening for underserved populations [24], few programs have focused on prostate cancer screening for males. The present study aims not only to describe the outcomes of the screening events, but also the process of collaborating with a community-based partner and the logistics of offering prostate cancer screening outside of a traditional clinic setting. The eight community events screened almost 300 males, many of whom were at increased risk of prostate cancer: 85% were Black males and 40% had not been screened in the past three years.

The success of the SKCCC MCSP can likely be attributed largely to the collaborative effort between community partners and the SKCCC Office of COE to increase awareness, educate, and bring screenings to historically underserved communities. Historical injustices within the healthcare system towards communities of color, particularly Black or African American individuals, has fostered a culture of mistrust between Black communities and healthcare systems [25, 26]. Community-based organizations (CBOs) are a foundational aspect of trust within historically underserved communities as their primary work is grounded within them. The partnerships between healthcare systems and CBOs such as those demonstrated within the COE team at the SKCCC work to not only increase access but build trust between medical institutions and underserved communities.

Among the males who were screened, Black males had the highest average PSA level. Although not statistically different than the males from other racial or ethnic groups, our finding replicates what has previously been documented in the literature about Black males having some of the highest PSA levels among males [27]. Individuals who had two or more relatives with a history of prostate cancer also had a significantly higher PSA level than those without relatives or only one relative with a history of prostate cancer. Both findings are consistent with data that shows that two of the most important risk factors are having a Black racial identity and a family history of prostate cancer [2]. These findings also demonstrate the effectiveness of the SKCC MCSU in reaching and screening individuals at a higher risk of developing prostate cancer.

Though community-based research is a critical step towards more equitable health care and trust-building, it poses many challenges. There are many logistical factors involved in bringing screening to different community locations which must be coordinated by all members of the screening team in collaboration with the CBO. To offer community-based prostate cancer screening, the SKCCC MCSP employs a phlebotomist who can attend each event. The team transports all supplies and materials to and from events, paying particular attention to the storage requirements of the blood samples and the privacy of the questionnaire data. A nurse partitioner from urology attends each event to be able to offer shared decision making to males who are considering prostate cancer screening. Community screening programs are an invaluable way to overcome barriers to routine cancer screening; however, it is important to acknowledge their intricacies and challenges.

There are limitations to this research that should be noted. The survey items were self-reported and not verified by medical chart review, as males were met in community settings outside of our health system. For some survey questions, there was a substantial amount of missing data, which contributed to a less complete representation of the population. For example, it is unknown whether certain questions about the participant’s family history were left blank because there was no history of disease or simply because the individual did not know their family history. Data suggest that many males do not know their family history when it comes to risk of prostate cancer [28], and additional measures should be taken by the health care professionals on site to facilitate conversations regarding family history. Second, we are unable to track the outcome for males who have abnormal screening results. All males received copies of their results through the mail, and we encouraged them to share their results with a primary care physician or a urologist, to continue the discussion about their personal risk of prostate cancer. Moving forward, the SKCCC MCSP intends to address both of these limitations by checking survey data for completeness at the event and investing in additional staffing and resources to follow up with males who have abnormal screening results.

## Conclusion

Due to the increased risk of prostate cancer in Black men, it is important to utilize interventions that increase access to cancer screening. Community-based screening is an evidence-based approach that reduces barriers to screening and builds relationships of trust between medical institutions and the community. Our results are consistent with previous data regarding elevated PSA levels in males who are Black or African American, and those with a family history of prostate cancer. Community-based screening requires greater logistical capacity than clinic-based screening programs; however, the SKCCC MCSP will continue to work towards an ever-evolving inclusive and far-reaching program to ultimately help reduce inequities in prostate cancer screening, diagnosis, and care.

## Data Availability

Data is available from the corresponding author upon request.
